# Untreated Injuries to the Anterolateral Capsular Structures Do Not Affect Outcomes and Kinematics after Anatomic Anterior Cruciate Ligament Reconstruction

**DOI:** 10.3390/jcm12134408

**Published:** 2023-06-30

**Authors:** Elmar Herbst, Joanna Costello, Adam J. Popchak, Scott Tashman, James J. Irrgang, Freddie H. Fu, Volker Musahl

**Affiliations:** 1 Department of Orthopaedic Surgery, University of Pittsburgh Medical Center, Pittsburgh, PA 15261, USA; 2 Department of Trauma-, Hand- and Reconstructive Surgery, University Hospital Muenster, 48149 Münster, Germany; 3 Department of Radiology, University of Pittsburgh Medical Center, Pittsburgh, PA 15261, USA; 4 Department of Physical Therapy, University of Pittsburgh, Pittsburgh, PA 15213, USA; 5 Steadman Philippon Research Institute, Vail, CO 81657, USA

**Keywords:** ACL, knee, rotatory knee laxity, pivot shift, in vivo, kinematics, outcome

## Abstract

Background: Injuries to the anterolateral complex (ALC) may contribute to increased rotatory knee laxity. However, it has not been evaluated whether such injuries affect in vivo kinematics when treated in situ. The purpose of this study was to determine the grade of ALC injury and its effect on kinematic and clinical outcomes of ACL-injured patients 24 months after anatomic ACL reconstruction. It was hypothesized that injury to the ALC would be significantly related to patient-reported outcomes (PROs) and in vivo knee kinematics during downhill running. Methods: Thirty-five subjects (mean age: 22.8 ± 8.5 years) participating in a randomized clinical trial to compare single- and double-bundle ACL reconstruction were included in the study. Subjects were divided into two groups based on the presence or absence of injury to the ALC, as determined on MRI scans performed within 6 weeks of injury. None of the patients underwent treatment for these ALC injuries. At 24 months, PROs, including the International Knee Documentation Committee Subjective Knee Form (IKDC-SKF), Knee injury and Osteoarthritis Outcome Score (KOOS) and in vivo knee kinematics during downhill running, were obtained. Pivot-shift test results, PROs and in vivo knee kinematics were compared between groups with and without ALC injury using the Pearson’s Chi Squared test and Mann–Whitney U test with significance set at *p* < 0.05. Results: The average interval between injury and performing the MRI scans was 9.5 ± 10 days. ALC injury was observed in 17 (49%) study participants. No significant differences were detected in PROs and in vivo kinematics between subjects with and without ALC injury (n.s.). Conclusion: The findings of this study demonstrate that MRI evidence of an ALC injury does not significantly affect in vivo knee kinematics and PROs even in individuals with a high-grade ALC injury. Injuries to the ALC as observed on MRI might not be a useful indication for an anterolateral procedure.

## 1. Introduction

Over the last several years, the anterolateral complex (ALC) of the knee and its effect on knee biomechanics and kinematics has been a topic of interest and debate [1,2,3,4,5]. While technical advancements in anterior cruciate ligament (ACL) surgery led to improved outcomes [6], some patients continue to have persistent rotatory knee laxity [7,8,9]. It is well known that excess anterolateral rotatory knee laxity, as evidenced by a positive pivot-shift test, might negatively affect patient-reported outcomes (PROs) [7,10].

While the pivot-shift test is influenced by multiple factors involving soft tissues and bone morphology [1,2,11,12,13], most of the recent research has been dedicated to the anterolateral knee structures only. However, conflicting reports have been published regarding the contribution of the anterolateral complex to rotatory knee laxity. While some authors found the anterolateral ligament (ALL) or anterolateral capsule to be an important restraint to internal tibial rotation [14,15,16], others report that it has a negligible role in controlling rotatory knee laxity, especially if the iliotibial band is intact [1,3,17,18]. Clinically, concomitant injuries to the anterolateral capsule in an ACL-deficient knee as observed on magnetic resonance imaging (MRI) have been shown to be associated with increased rotatory knee laxity [2,19]. Thus, lateral extra-articular procedures have been increasingly proposed to properly address this anterolateral rotatory laxity [9,20,21]. However, only a few studies investigated the healing capacity as well as the clinical consequences of ALC injuries treated in situ and only one of these studies reported on in vivo kinematics following ACL reconstruction with or without an anterolateral tenodesis in subjects with ALC injuries [22,23,24,25]. Clinical evidence suggests that even an avulsion of the ALC, a Segond fracture, does not negatively affect clinical outcomes [26].

To better understand the effect of concomitant injuries to anterolateral capsular structures treated in situ, the in vivo knee kinematics and PROs of these patients might provide valuable information to further identify the indications for a lateral extra-articular procedure. Thus, the purpose of this study was to determine the effect of injury to the ALC on kinematic and patient-reported outcomes of ACL-injured patients 24 months after anatomic ACL reconstruction. It was hypothesized that injury to the ALC, ranging from edema to complete disruption, determined on MRI scans would significantly affect PROs as well as in vivo knee joint kinematics during downhill running 24 months after ACL reconstruction.

## 2. Materials and Methods

This is a secondary analysis of data collected during a prospective randomized controlled trial designed to compare single- vs. double-bundle ACL reconstruction. The inclusion and exclusion criteria of that trial have been described elsewhere [27]. Following institutional review board approval (PRO09020493) and written informed consent, 47 subjects (32 males, 15 females; mean age: 22.0 ± 7.6 years) with unilateral ACL injury were included. For this secondary analysis, patients with incomplete data (in vivo kinematics for both knees, PROs) or a time interval of more than 6 weeks between injury and initial MRI were excluded to evaluate the effects of injury to the ALC, leaving 35 subjects (mean age: 22.8 ± 8.5 years) for evaluation.

### 2.1. Surgical Procedure and Rehabilitation

Before ACL reconstruction, an examination under anesthesia including the pivot-shift test was performed by experienced knee surgeons who were blinded to MRI results. The pivot-shift test was graded according to the International Knee Documentation Committee (IKDC) knee ligament evaluation guidelines and rated as low grade (negative or 1+) or high grade (2+ and 3+). Individualized anatomic ACL reconstruction (either single- or double-bundle) was performed using a 10 mm quadriceps tendon autograft with a bone block [28]. For double-bundle ACL reconstruction, a split graft was used with the bone block in one femoral tunnel. The two arms of the tendon were placed in separate tunnels created in the center of the tibial anteromedial (AM) and posterolateral (PL) bundle insertions. For femoral graft fixation, an extra-cortical button with a continuous loop was used. Tibial fixation occurred at full extension for the PL bundle and at 45° for the AM bundle using interference screws. For single-bundle ACL reconstruction, the bone tunnels were located in the center of the native femoral and tibial footprints and graft fixation occurred at 20° of knee flexion. Postoperatively, the same standard rehabilitation protocol was applied to all patients.

### 2.2. In Vivo Joint Kinematics

Three-dimensional in vivo kinematics data were acquired using dynamic stereo X-ray (DSX) during downhill running (3.0 m/s, 10° downward slope) on an instrumented dual-belt treadmill (Bertec Corp, Columbus, OH, USA) 24 months after ACL reconstruction. Each participant performed the downhill run three times for each leg wearing running shoes.

The DSX system consisted of two gantries containing 100 kW pulsed X-ray sources (CPX 3100CV; EMD Technologies, Inc., Saint-Eustache, QC, Canada) and detectors. The gantries were positioned so that the X-ray beams intersected at 60° in a plane parallel to the floor. Image acquisition occurred at 150 frames/s with 90 kVp and 125 mA.

Kinematics data were collected shortly before heel strike, as determined by the vertical ground reaction force from the treadmill, through to the early stance phase (first 10% of a full gait cycle) as previously described in detail [29,30].

High-resolution computed tomography (CT) scans (0.31 × 0.31 × 0.6 mm voxels) were obtained from both knees six months after ACL reconstruction to determine bone geometry. These CT bone models were matched over the stereoscopic images using a previously validated model-based tracking process to determine tibiofemoral motion from the DSX images [30]. In brief, using a 12-marker phantom and a direct linear transformation calibration algorithm, the bone geometry of the DSX system was determined. Tibiofemoral models were produced by manually segmenting the CT scans using Mimics (Materialize, Inc., New York, NY, USA). The model-based tracking has been shown to have an accuracy of 0.6 ± 0.3° for dynamic external–internal rotation and 0.3 ± 0.1° for dynamic abduction–adduction [30]. Tibiofemoral motion was calculated from initial ground contact through the first 10% of the gait cycle as previously described [29]. Six-degree-of-freedom rotations of the tibia relative to the femur were calculated based on the conventions proposed by Grood and Suntay [31].

For the present study, side-to-side difference (SSD) of peak internal tibial rotation, range of internal–external tibial rotation, peak adduction, range of abduction–adduction, peak anterior tibial translation and range of anterior–posterior tibial translation were included in the final analysis. These variables were selected based on the proposed function of the ALC.

### 2.3. MRI Analysis and Patient-Reported Outcome Measures

**Figure 1:**
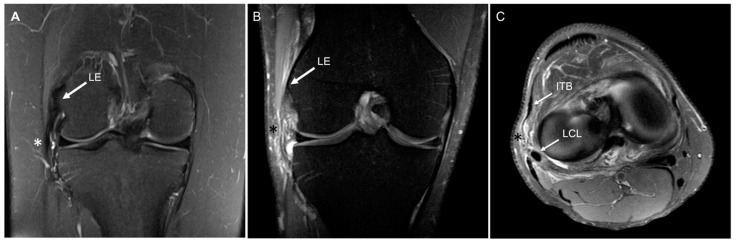
T2 fat suppressed magnetic resonance imaging scans from a right knee with (**A**) no sign of an anterolateral complex injury on the coronal view (white asterisk). In (**B**,**C**), a grade 2 anterolateral complex injury with partial fiber disruption and edema is visible as indicated with the black asterisk. LE, lateral epicondyle; LCL, lateral collateral ligament; ITB, iliotibial band.

Preoperative MRI scans using a 1.5 T open-bore magnet and a slice thickness 3 mm (Signa; GE Healthcare, Chicago, IL, USA) were retrospectively screened for evidence of injury to the ALC by a fellowship trained musculoskeletal radiologist who was blinded to the PROs and in vivo joint kinematics. Injuries to the ALC were classified based on T2-weighted fat suppression images as previously described [2]. Briefly, grade 1 signals were characterized by minimal intrinsic and/or adjacent edema, grade 2 injuries had edema within and surrounding the capsule with partial fiber disruption, whereas grade 3 injuries had complete disruption of fibers (Figure 1). 

Patient-reported outcomes were collected 24 months following ACL reconstruction and included the International Knee Documentation Committee Subjective Knee Form (IKDC-SKF) [32] and the Knee injury and Osteoarthritis Outcome Score (KOOS) [33]. At 24 months following ACL reconstruction, anterior tibial translation as measured using the KT-1000 arthrometer (MEDmetric Corp, San Diego, CA, USA) and anterolateral rotatory knee laxity as tested using the pivot-shift test were recorded by experienced knee surgeons, who were blinded to MRI results. Grading of the pivot-shift test was based on the convention of the IKDC Knee Ligament Rating Guidelines.

### 2.4. Statistical Analysis

For statistical analysis, SPSS version 27 (SPSS Inc., Chicago, IL, USA) was used. Subjects were dichotomized into two groups based on the presence or absence of injury to ALC determined on MRI. For a second analysis, patients were dichotomized into two groups based on whether ALC fiber disruption was evident on MRI (grade 2 and 3) or not (grade 0 and 1). Quantitative parameters are expressed as means ± one standard deviation (SD). Laxity measurements were compared between the two groups with Pearson’s Chi Square test and the Mann–Whitney U test was used for the between-group comparisons for the PROs and in vivo kinematics. Statistical significance was set at *p* < 0.05.

## 3. Results

Preoperative MRIs were obtained on average 9.5 ± 10.1 days after ACL injury. Evaluation of the ALC revealed an injury in seventeen (49%) subjects. Of these seventeen patients, six had a grade 1 injury, ten had a grade 2 injury and one had a grade 3 injury.

All patients with concomitant radiographic ALC injury had a low-grade pivot shift as determined at the time of the examination under anesthesia prior to ACL reconstruction. In contrast, 7 of 18 patients in the group without MRI evidence of ALC injury had a grade 2 or 3 pivot shift, which was significantly more frequent when compared to those subjects with an ALC injury (*p* < 0.05).

**Figure 2:**
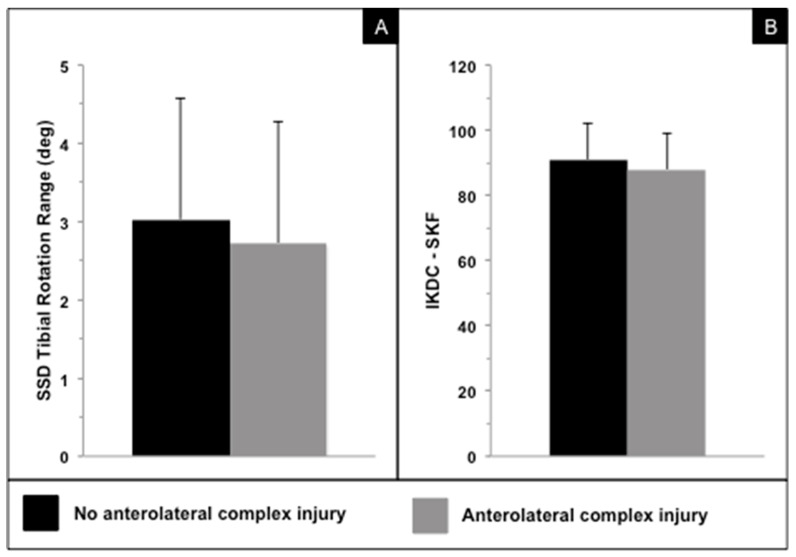
(**A**) In vivo side-to-side difference (SSD) of internal–external tibial rotation range in degrees during downhill running in patients with and without concomitant anterolateral complex injury. (**B**) International Knee Documentation Committee Subjective Knee Form (IKDC-SKF) in patients with and without concomitant anterolateral complex injury. No significant differences were observed for in vivo knee kinematics and patient-reported outcomes.

Twenty-four months following ACL reconstruction, there were no significant differences in in vivo kinematics and PROs between patients who had an ALC injury and those who had no MRI evidence of ALC injury (*p* > 0.05) (Figure 2, Table 1 and Table 2). Notably, the side-to-side difference (SSD) in the peak internal tibial rotation displayed more external tibial rotation following ACL reconstruction in the affected knee when compared to the healthy contralateral knee (Table 1). Further, subjects who had a concomitant ALC injury on MRI at time zero had a more externally rotated tibia as compared to the contralateral healthy knee by on average 1.6° during downhill running at final follow-up (Table 1). Table 1 and Table 2 summarize the results for in vivo kinematics and IKDC-SKF and KOOS subscales.

When comparing patients who had evidence of partial or complete ALC fiber disruption on MRI (grade 2 and 3) with those who had no injury or only edema in the area of the ALC (grade 0 and 1), no significant differences in terms of PROs and in vivo knee joint kinematics were observed (all *p* > 0.05).

At 24 months follow-up, only one patient in the ALC injury group had a grade 1 pivot shift compared to no patients in the group without MRI evidence of ALC injury (*p* = 0.309). Comparing SSD in anterior tibial translation as measured using the KT-1000 arthrometer, those subjects with a grade 2 and 3 ALC injury displayed significantly more anterior tibial translation compared to subjects with a low-grade ALC tear (1.6 ± 0.8 mm vs. 0.5 ± 0.3 mm; *p* = 0.008).

## 4. Discussion

The data from this study show that MRI evidence of an ALC injury at time zero does not significantly affect dynamic in vivo knee kinematics during downhill running and PROs 24 months after ACL reconstruction, even if only higher-grade ALC disruptions are considered. The data indicate that injuries to the ALC treated in situ do not adversely affect functional outcomes following ACL reconstruction. In the present study, 49% of patients had MRI evidence of an ALC injury ranging from edema to complete fiber tear. However, only one subject displayed a complete capsular fiber disruption, whereas only edema or partial fiber disruption were observed in the other sixteen patients. In a previous study, the frequency of such signal alterations in the ALC, as observed on MRI in the setting of a complete ACL injury, was comparable (51%) [2]. A recent study investigating the visibility of ALL tears on 1.5 T MRIs revealed a poor intra- and inter-observer reliability in detecting such injuries [34]. Since in the current study surgical exploration of the ALC was not performed, the authors are not able to report on the proportion of visible ALC injuries at the time of ACL surgery. Studies exploring the anterolateral knee structures surgically described the presence of a visible injury in up to 93% of the cases [35,36]. However, most of these injuries involved hemorrhage only, whereas complete disruption of the ALC was less frequent [36], which is consistent with the MRI findings in the present study. 

In contrast to previous studies [2,37], ALC injuries were not associated with a higher pivot-shift grade. However, the extent of an anterior translation of the lateral knee compartment during the manual pivot-shift test was not quantified [2,37]. Thus, the pivot-shift results in the present study are based on manual grading by experienced knee surgeons, who were, however, at the time of surgery and final follow-up, blinded to the MRI results, which were evaluated in retrospect.

Dynamic in vivo kinematics during downhill running revealed no statistically significant differences in terms of side-to-side differences in tibial rotation as well as knee abduction and anterior tibial translation between subjects with an ALC injury and those without any ALC tear. Interestingly, in both groups, the tibia of the involved knee was more externally rotated after ACL reconstruction when compared to the contralateral healthy knee. These findings are consistent with the vast majority of the studies investigating dynamic, functional knee kinematics, where ACL reconstruction was shown to result in an increased external tibial rotation when compared to the uninvolved contralateral knee [38,39,40]. Considering that tibial rotation was not significantly affected by an injury to the ALC, any additional lateral extra-articular procedure might result in a greater external rotation of the tibia. However, it should be mentioned that the task completed during the collection of the kinematic data in this study (downhill running) was not a task that produces high rotatory knee torques. Therefore, the question remains if such highly demanding tasks would adversely affect the results of the present study.

Some studies on cadaveric knees reported the importance of the ALL or ALC in restraining internal tibial rotation [14,15,16,37]. However, one should consider that either a large incision of the ALC [14,37] was performed or the iliotibial band was resected in these studies [15,16]. Such a large tear of the ALC, however, does not reflect the typical injury pattern of a patient with an ACL tear commonly seen in daily clinical routine. Thus, biomechanical studies may overestimate the effect of ALC injuries on tibial rotation. Further, it has been shown that the iliotibial band and its deeper layers are the strongest ALC structures and provide the greatest restraint to internal tibial rotation, whereas the anterolateral capsule behaves more like a sheet of tissue rather than a true ligament [1,3,18,41]. When the ALC displays an injury on MRI, with the iliotibial band being intact, knee kinematics might not be relevantly affected. Further, it could also be that such interstitial ALC injuries might heal over time when the iliotibial band and ACL reconstruction provide sufficient knee stability, as data from Segond avulsion may suggest [26].

In contrast, clinical data support the use of an anterolateral procedure to reduce the risk of ACL graft failures, especially in young patients returning to high-risk pivoting sports [5,42]. Nevertheless, an overconstraint of knee kinematics may remain a concern as long-term data regarding modern combined ACL reconstructions with anterolateral procedures are still pending [25,43,44].

There were no differences in PROs between the two groups of interest, even if all obtained scores were slightly lower in those patients with the presence of an ALC injury. Overall, however, the PROs were comparable to those commonly seen following isolated ACL reconstruction [45] or combined ACL and anterolateral surgery [5,9].

The limitations of this study include the functional task performed for the evaluation of dynamic in vivo kinematics (downhill running). Further, this study does not provide data on the natural history of ALC injuries. Another limitation includes the fact that this is a secondary analysis of a randomized trial comparing single- vs. double-bundle ACL reconstruction. As recently reported, there were not significant differences in PROs and in vivo kinematics between the two surgical techniques within this cohort [40,46]. Additionally, the follow-up of two years includes short-term data only. Thus, this study does not provide any insights on the long-term consequences of untreated anterolateral injuries. Long-term data are however needed to further understand whether such untreated anterolateral injuries or lateral extra-articular procedures affect ACL graft integrity, knee kinematics and altered tibiofemoral contact forces.

Even if the current study did not detect significant differences between patients with and without anterolateral injuries, based on the current literature surgeons should consider the amount of rotatory knee laxity, bone morphology (i.e., posterior tibial slope), meniscal integrity (i.e., posterolateral meniscal root tears) and generalized hyperlaxity when opting for an additional anterolateral procedure.

## 5. Conclusions

The findings of this study demonstrate that MRI evidence of an ALC injury does not significantly affect in vivo knee kinematics or patient-reported outcome scores. Thus, based on these findings, performing additional lateral extra-articular procedures to reduce excess rotatory laxity due to presence of ALC injury is not supported. Accordingly, it is recommended to observe MRI-detectable injuries to the ALC as well as other features known to be associated with the pivot-shift phenomenon (i.e., posterior tibial slope) to identify the cause of an increased (residual) rotatory knee laxity.

## Figures and Tables

**Table 1:** In vivo knee kinematics.

	No ALC Injury	ALC Injury	*p*-Value
	Mean (SD)	Mean (SD)	
Side-to-side difference			
Peak internal tibial rotation (deg)	0.3 (3.3) *	1.9 (3.2) *	0.310
Internal–external tibial rotation range (deg)	3.0 (1.5)	2.7 (1.6)	0.443
Peak adduction (deg)	0.6 (1.0) **	0.8 (1.3) **	0.663
Abduction–adduction range (deg)	1.0 (0.6)	0.9 (0.8)	0.917
Peak anterior tibial translation (mm)	2.0 (2.4) ***	0.6 (2.8) ***	0.436
Anterior–posterior tibial translation range (mm)	3.7 (2.3)	5.0 (3.3)	0.233

* Refers to greater external tibial rotation when compared to the healthy contralateral knee; ** refers to greater adduction when compared to the healthy contralateral knee; *** refers to greater anterior tibial translation when compared to the healthy contralateral knee.

**Table 2:** Patient-reported outcomes.

	No ALC Injury	ALC Injury	*p*-Value
	Mean (SD)	Mean (SD)	
IKDC—SKF	91.0 (10.9)	88.0 (11.2)	0.348
KOOS—Symptoms	89.8 (7.7)	84.5 (11.1)	0.181
KOOS—Pain	95.7 (6.6)	93.1 (13.6)	0.775
KOOS—ADL	99.1 (2.1)	96.9 (11.3)	0.976
KOOS—Sport/Rec	91.3 (13.4)	90.3 (11.7)	0.620
KOOS—QOL	87.9 (17.6)	77.5 (24.8)	0.169

## Data Availability

Data available on request due to restrictions e.g., privacy or ethical. The data presented in this study are available on request from the corresponding author. The data are not publicly available due to university specific data policies.
